# Tetrathionate sensing enables *Vibrio parahaemolyticus* to repress quorum sensing for intestinal colonization

**DOI:** 10.1080/19490976.2026.2748145

**Published:** 2026-10-15

**Authors:** Xiaojun Zhong, Xiujuan Zhou, Mengting Shi, Tianqi Liang, Yuanyang Xu, Kexin Gu, Huochun Yao, Menghua Yang

**Affiliations:** a College of Animal Science and Technology, College of Veterinary Medicine, Zhejiang A & F University, Key Laboratory of Applied Technology on Green-Eco-Healthy Animal Husbandry of Zhejiang Province, Zhejiang Provincial Engineering Laboratory for Animal Health Inspection & Internet Technology, Hangzhou, China; b MOE Joint International Research Laboratory of Animal Health and Food Safety, College of Veterinary Medicine, Nanjing Agricultural University, Nanjing, China; c Basic Medical and Forensic Medicine College, Hangzhou Medical college, Hangzhou, China

**Keywords:** *Vibrio parahaemolyticus*, tetrathionate, quorum sensing, two-component system, intestinal colonization

## Abstract

Quorum sensing (QS) enables bacterial coordination of group behaviors, yet how enteric pathogens modulate QS during intestinal colonization remains poorly understood. Here, we demonstrated that *Vibrio parahaemolyticus* represses QS to promote gut colonization through sensing of self‑generated tetrathionate derived from host thiosulfate. We found that the cecal extracts from healthy mouse contained thiosulfate, which could be converted to tetrathionate by *V*. *parahaemolyticus* via the thiosulfate dehydrogenase TsdBA. The two‑component system TtrRS then sensed this self‑produced tetrathionate and, via the global silencer H‑NS, repressed expression of the master QS regulator OpaR independently of the canonical QS receptors. Transcriptomic analyzes initially uncovered a cross‑talk between TtrRS and OpaR, showing co‑regulation of 76 genes with opposing patterns, and further study demonstrated that OpaR directly binds and represses the *tsdBA* promoter, establishing a coherent regulatory loop in which TtrRS and OpaR converge on shared target genes. Deletion of *opaR* significantly enhanced bacterial colonization in a mouse model, indicating that QS actively restricts intestinal fitness of *V*. *parahaemolyticus*, a restriction that is relieved by TtrRS‑mediated QS repression. Collectively, *V*. *parahaemolyticus* exploits TsdBA‑dependent tetrathionate generation to downregulate QS through a TtrRS-H-NS-OpaR cascade, with cross‑talk between TtrRS and OpaR fine‑tuning this adaptive response, thereby conferring a competitive advantage in the host gut. These findings provide a new model for how enteric pathogens integrate sulfur metabolism and QS repression to optimize host colonization.

## Introduction

Bacteria exhibit a high degree of intercellular communication, in which quorum sensing (QS) plays a central role.^1^ Through the production and detection of extracellular chemical signal molecules known as autoinducers (AIs), QS enables bacteria to coordinate group behaviors such as bioluminescence, motility, biofilm formation, and virulence in many pathogens.^1^ ^,^ ^2^ However, the fitness advantage conferred by QS activation is highly dependent on environmental context, and in some niches, QS repression may instead favor bacterial adaptation.^3^ ^,^ ^4^ AIs produced by different bacterial species can be recognized to varying extents, thereby helping to maintain microbial homeostasis within specific ecological niches.^1^ ^,^ ^2^ In the host, particularly the intestinal tract, a vast and diverse microbial community resides, producing a wide array of AIs and metabolites. Nevertheless, the mechanisms by which host- and microbiota-derived metabolites influence the QS activity of enteric pathogens during infection remain largely unexplored.

During host colonization, pathogens often exploit external signals to modulate gene expression and promote infection. For example, *Vibrio cholerae* senses host dietary fatty acids via the membrane-associated transcription regulators TcpP and TcpH to induce expression of the virulence factor ToxT.^5^ Uropathogenic *Escherichia coli* uses the two-component system (TCS) GrpP/GrpQ to respond to D-serine in urine, thereby activating type 1 fimbria expression and enhancing pathogenicity.^6^ Such regulatory systems are conserved and versatile signal transduction modules that enable bacteria to sense specific environmental cues and adapt to hostile conditions.^7^ ^,^ ^8^ Bacteria employ multiple signaling systems that can function independently or cross-talk, forming complex regulatory networks.^9^ ^,^ ^10^ However, how pathogens integrate host-specific signals to orchestrate cross-talk among distinct signaling pathways for infection modulation remains poorly understood.

*Vibrio parahaemolyticus* is a major foodborne pathogen that causes acute gastroenteritis in humans.^11-13^ With ongoing global warming and increasingly complex food supply chains, infections caused by *V*. *parahaemolyticus* are becoming more frequent not only in coastal regions but also in inland cities.^13-15^ This bacterium is typically found in warm marine or estuarine environments.^16^ ^,^ ^17^ Upon entry into the human gastrointestinal tract, it encounters a variety of extreme conditions and must efficiently perceive environmental changes and reprogram gene expression to adapt to the host niche. Both TCSs and QS have been shown to regulate multiple physiological processes in *V*. *parahaemolyticus.* ^18-23^ Nonetheless, the signaling pathways mediated by these regulators have rarely been linked to environmental signals, and how *V*. *parahaemolyticus* senses host-derived cues in the gut to control colonization is still largely undefined.

In this study, we demonstrated that *V*. *parahaemolyticus* utilizes the TCS TtrRS to sense the self‑generated tetrathionate derived from host thiosulfate. This sensing event represses QS activity through a previously unrecognized pathway, thereby promoting bacterial colonization in the host gut. Our findings provide new insights into how bacterial pathogens exploit host-specific signals to achieve cross-talk between different signaling systems for infection regulation, and they may offer a theoretical basis for developing novel antimicrobial strategies that target multiple signaling pathways, contributing to the control of intestinal bacterial infections.

## Results

### Host intestinal molecules repress quorum sensing of *V*. *parahaemolyticus*

The mammalian intestinal tract harbors a complex milieu of host-derived molecules and microbial metabolites that enteric pathogens must sense and adapt to for successful colonization.^24^ ^,^ ^25^ To determine whether intestinal molecules influence QS in *V*. *parahaemolyticus*, we harvested luminal contents from the cecum and small intestine and incubated them with a wild-type (WT) *V*. *parahaemolyticus* strain harboring a QS reporter plasmid (Figure 1A). This reporter contained the bioluminescence *luxCDABE* operon cloned from *Vibrio harveyi.* ^22^ Measurement of bioluminescence production revealed that both cecal and small intestinal contents inhibited the luminescence signal of the P_*vh*_-*lux* reporter in a dose-dependent manner (Figure 1B). To corroborate this observation, we transformed the WT strain with an independent reporter plasmid, P_*opaR*_-*lux*, which carries the promoter region of the QS master regulator *opaR*. Consistent with the previous results, both cecal and small intestinal contents suppressed P_*opaR*_-*lux* luminescence in a dose-dependent fashion (Figure 1C). Collectively, these findings indicate that host intestinal molecules are sensed by *V*. *parahaemolyticus* to modulate QS activity.

**Figure 1.**
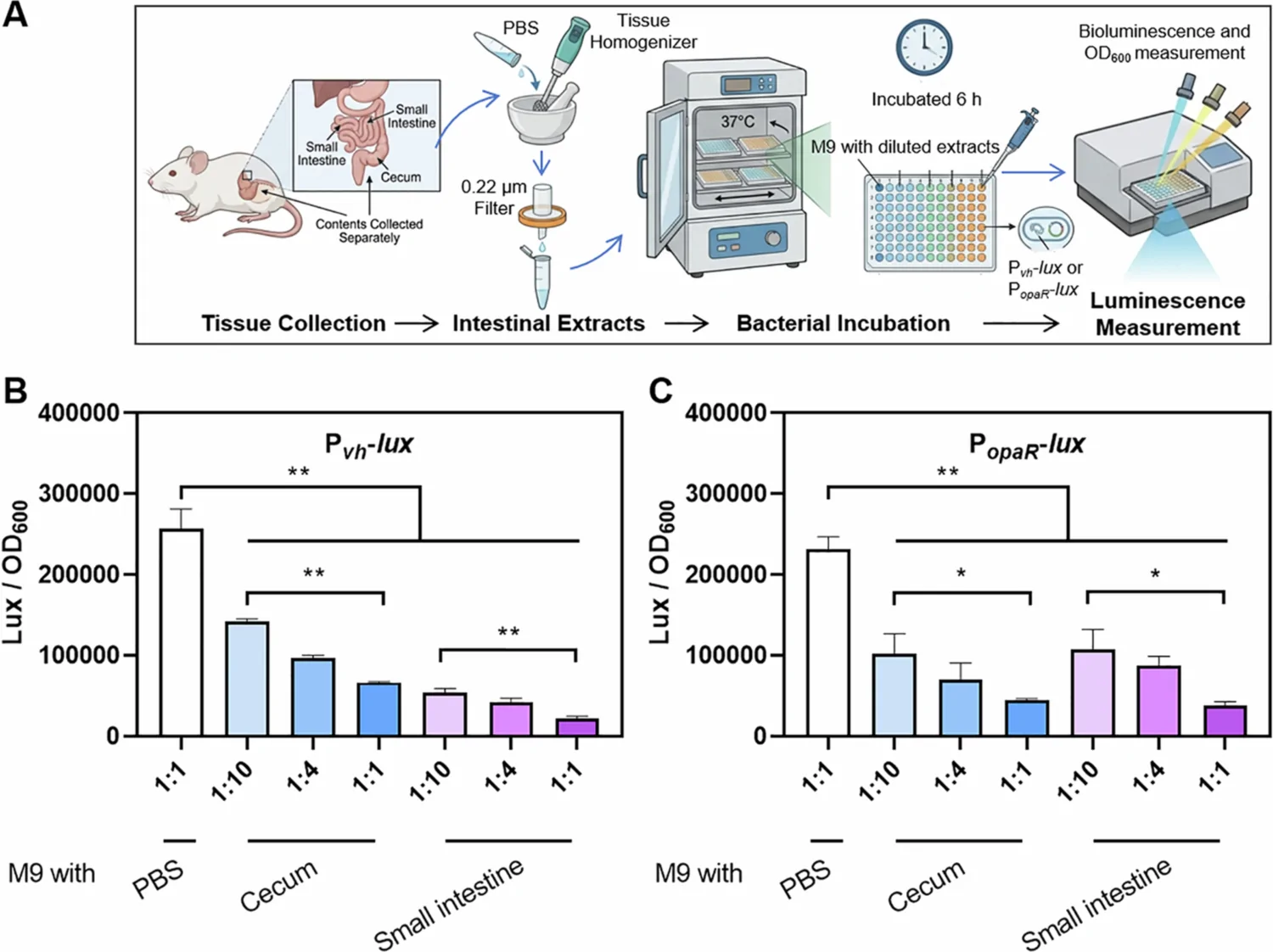
Host intestinal contents repress QS activity in *V. parahaemolyticus* in a dose‑dependent manner. (**A**) Flowchart of the experimental procedure for assessing the effect of intestinal contents on QS activity. Cecal and small intestinal luminal contents were collected from mice, homogenized, centrifuged, and filter‑sterilized to obtain intestinal extracts. Wild‑type *V*. *parahaemolyticus* harboring a QS reporter plasmid (P_*vh*_‑*lux* or P_*opaR*_‑*lux*) was incubated with serial dilutions of the extracts. Luminescence production was measured as an indicator of QS activity. (**B, C**) Effects of cecal and small intestinal luminal contents on QS activity. *V*. *parahaemolyticus* harboring P_*vh*_‑*lux* or P_*opaR*_‑*lux* was incubated with increasing concentrations of cecal or small intestinal contents. Luminescence expression was calculated as the luminescence per unit of OD_600_ after 6 h of incubation at 37 °C. The one-way ANOVA followed by Tukey’s test was used for statistical analysis (*, *P* < 0.05; **, *P* < 0.01).

### Intestinal molecules repress quorum sensing independently of canonical autoinducer receptors in *V*. *parahaemolyticus*

The QS circuitry of *V*. *parahaemolyticus* comprises AI synthases (LuxM, CqsA, and LuxS), membrane-bound receptors (LuxN, CqsS, and LuxPQ), a two-component phosphorelay protein (LuxU/LuxO), and downstream master regulators (OpaR and AphA) (Figure 2A).^22^ ^,^ ^26^ At high cell density, signal transduction proceeds as high concentrations of AIs bind their cognate receptors, leading to the failure of LuxU/LuxO phosphorylation and thus the absence of Qrr1-5 transcription; as a result, the master regulator OpaR is relieved from repression and abundantly expressed to regulate diverse QS-associated physiological processes (Figure 2A).^22^ To investigate which components of this QS apparatus mediate the repressive effect of host intestinal molecules, we introduced the QS reporter plasmid P_*vh*_-*lux* into mutants lacking the three AI receptors (Δ*cqsS*Δ*luxN*Δ*luxPQ*; hereafter termed Δ3AI receptors) and into a *luxO* deletion mutant (Δ*luxO*). Strikingly, both cecal and small intestinal contents still inhibited P_*vh*_-*lux* luminescence in both mutant backgrounds (Figure 2B). To verify this finding with an independent QS reporter, we transformed the P_*opaR*_-*lux* plasmid into the same mutants. Consistent with the above results, both cecal and small intestinal contents suppressed P_*opaR*_-*lux* luminescence in the Δ3AI receptors and Δ*luxO* strains (Figure 2C). Collectively, these data demonstrate that host intestinal molecules are sensed by *V*. *parahaemolyticus* to repress QS through a pathway that operates independently of the canonical AI receptors.

**Figure 2.**
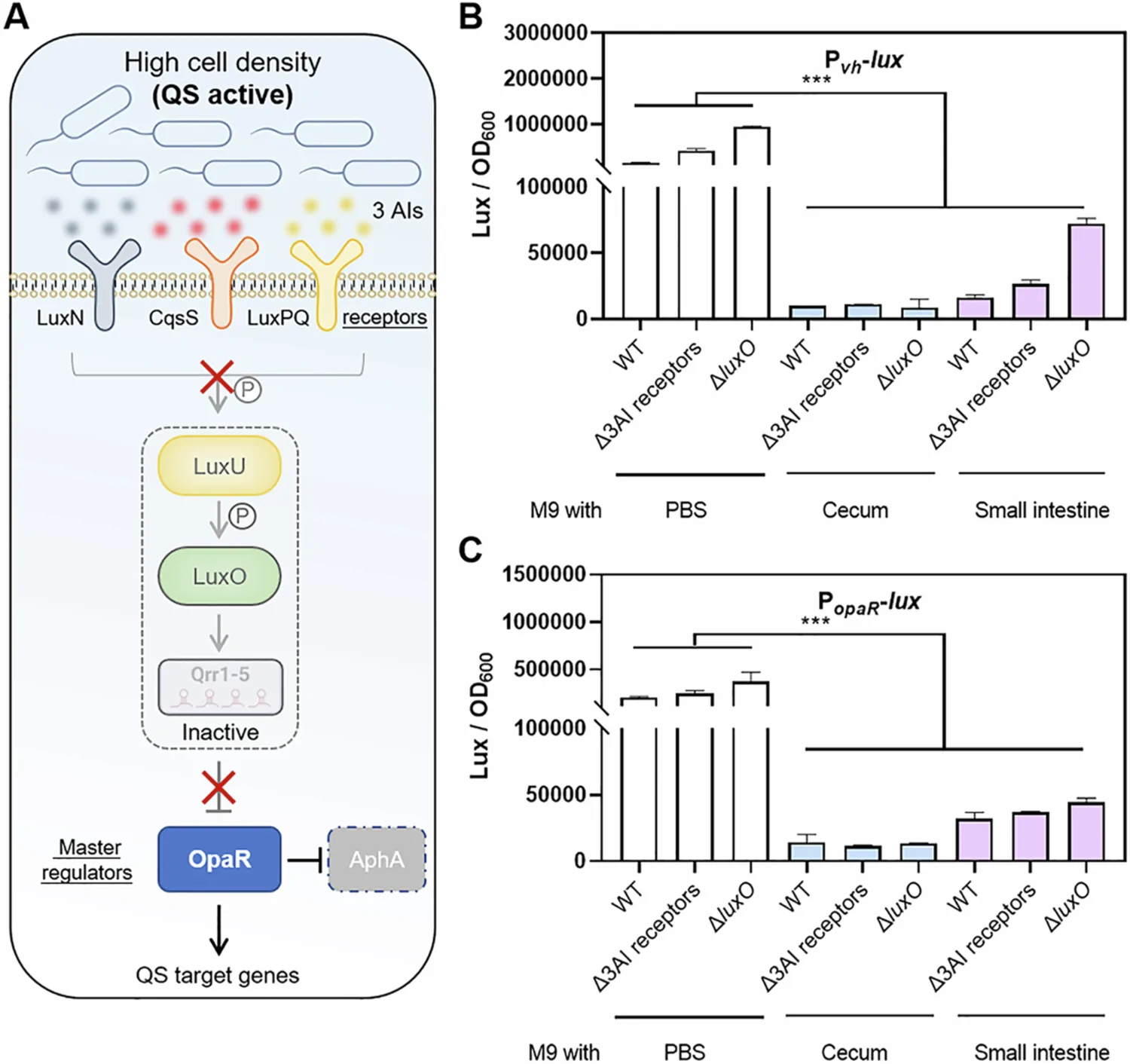
Host intestinal contents repress QS independently of the canonical AI receptors. (**A**) Schematic representation of the QS cascade in *V*. *parahaemolyticus* at high cell density. At this stage, high concentrations of AIs bind to their cognate membrane receptors (LuxN, CqsS, and LuxPQ), which consequently function as phosphatases rather than kinases. As a result, the phosphorelay protein LuxU and its downstream effector LuxO are maintained in their unphosphorylated state, preventing transcription of the small regulatory RNAs Qrr1‑5. Consequently, the master regulator OpaR is relieved from repression and abundantly expressed, thereby regulating diverse QS‑associated physiological processes. (**B, C**) Effects of cecal and small intestinal luminal contents on QS activity. WT, Δ3AI receptors (Δ*cqsS*Δ*luxN*Δ*luxPQ*), and Δ*luxO* strains carrying the P_*vh*_‑*lux* (B) or P_*opaR*_‑*lux* (C) were incubated with cecal or small intestinal contents. Luminescence expression was calculated as the luminescence per unit of OD_600_ after 6 h of incubation at 37 °C under high‑cell‑density conditions. The one-way ANOVA followed by Tukey’s test was used for statistical analysis (***, *P* < 0.001).

### The two-component system TtrRS senses tetrathionate generated from host-derived thiosulfate to repress quorum sensing in *V*. *parahaemolyticus*

Two-component systems (TCSs) are key signal transduction modules that enable bacteria to respond to environmental cues by regulating target gene expression, and typically consist of a membrane-bound histidine protein kinase (HK) and a cytoplasmic response regulator (RR).^27-29^ To rapidly identify TCSs involved in QS modulation in *V*. *parahaemolyticus*, we individually deleted 18 predicted RRs genes and examined the resulting mutants for altered QS activity in MLB medium. Several mutants exhibited significant changes in P_*vh*_-*lux* luminescence relative to the WT strain; notably, the Δ*ttrR* mutant displayed the highest luminescence among all tested strains (Figure S1). We have previously reported that the TtrRS TCS senses environmental tetrathionate and subsequently activates its target genes *ttrBCA*, and *tsdBA*, thereby contributing to intestinal colonization by *V*. *parahaemolyticus* (Figure 3A).^20^ To further investigate the role of TtrRS in QS regulation, we introduced the P_*vh*_-*lux* and P_*opaR*_-*lux* reporters into Δ*ttrR* and Δ*ttrA* mutant strains, and measured bioluminescence production under varying concentrations of tetrathionate. In the WT strain, tetrathionate inhibited QS activity in a dose-dependent manner; however, this inhibition was lost in the Δ*ttrR* strain (Figure 3B,C). We have previously demonstrated that TtrA is essential for tetrathionate reduction, and a Δ*ttrA* strain fails to consume tetrathionate.^20^ Here, we observed that although tetrathionate still suppressed QS activity in a dose-dependent manner in the Δ*ttrA* strain, the absolute luminescence levels of the QS reporters in this mutant were significantly lower than those in the WT strain (Figure 3B,C). Collectively, these results indicate that TtrRS senses tetrathionate to repress QS in *V*. *parahaemolyticus*.

**Figure 3.**
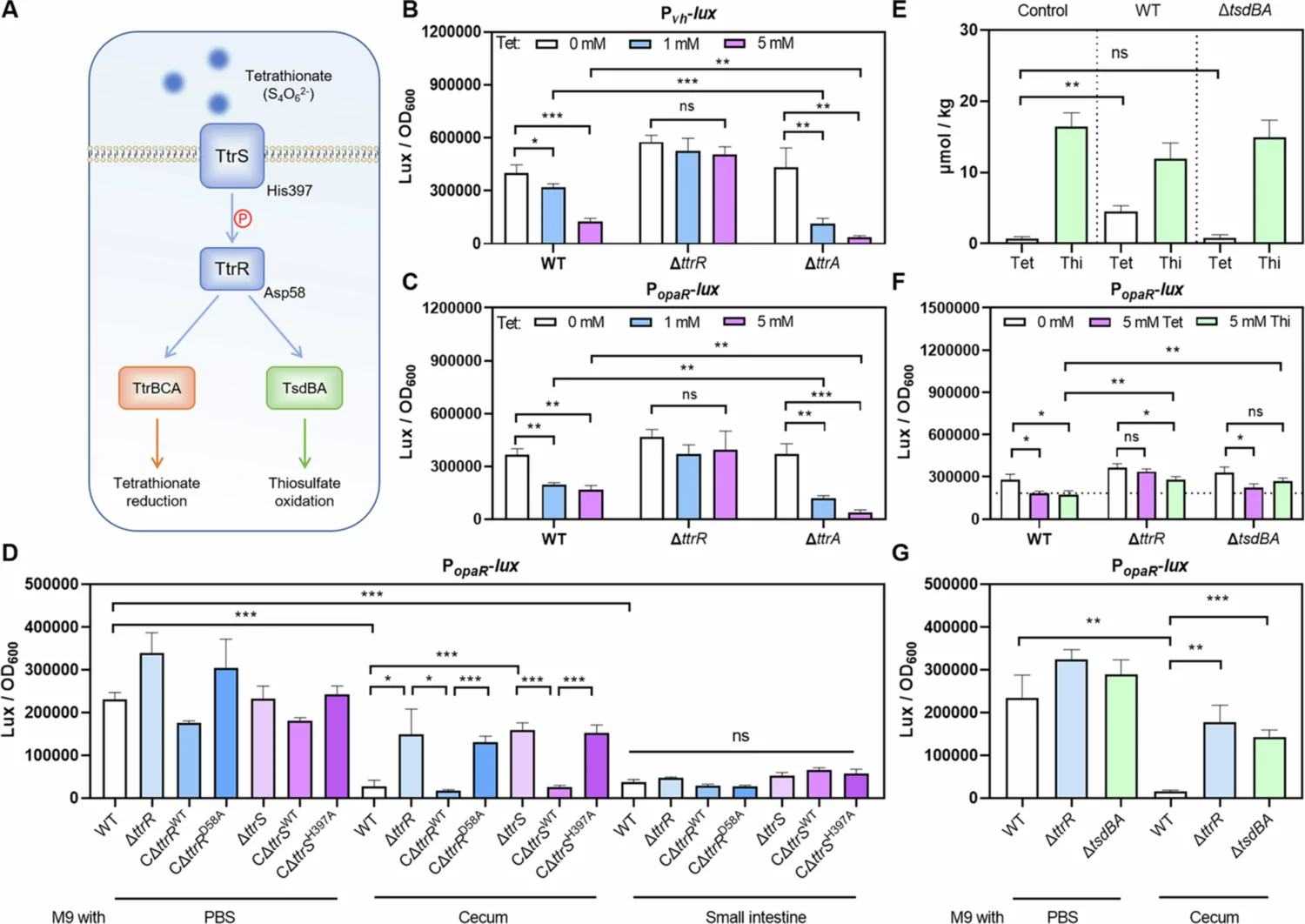
TtrRS senses tetrathionate to repress QS in *V. parahaemolyticus*. (**A**) Schematic model of the TtrRS signaling pathway. TtrS (membrane‑bound histidine kinase) senses environmental tetrathionate and phosphorylates TtrR (cytoplasmic response regulator) at residue Asp58 via its His397. Activated TtrR then induces expression of target genes *ttrBCA* and *tsdBA*, which contribute to intestinal colonization. (**B, C**) Effects of tetrathionate on QS activity in WT, Δ*ttrR*, and Δ*ttrA* strains. Strains carrying either the P_*vh*_‑*lux* or P_*opaR*_‑*lux* reporter were incubated with increasing concentrations of sodium tetrathionate for 6 h at 37 °C. (**D**) Cecal content‑mediated QS repression requires TtrRS phosphorylation activity. P_*opaR*_‑*lux* luminescence was measured in WT, deletion mutants (Δ*ttrR*, Δ*ttrS*), complemented strains (CΔ*ttrR*, CΔ*ttrS*), and strains carrying point mutations in the phosphorylation sites (TtrR^D58A^ or TtrS^H397A^) after exposure to cecal or small intestinal luminal contents. (**E**) Concentrations of thiosulfate and tetrathionate in mouse cecal extracts before and after incubation with WT and Δ*tsdBA* strains. (**F**) Effects of exogenous tetrathionate and thiosulfate on P_*opaR*_‑*lux* reporter activity in WT, Δ*ttrR*, and Δ*tsdBA* strains. (**G**) Effects of cecal luminal contents on P_*opaR*_‑*lux* reporter activity in WT, Δ*ttrR*, and Δ*tsdBA* strains. Luminescence expression was calculated as the luminescence per unit of OD_600_. The unpaired two-tailed Student’s *t*-test or the one-way ANOVA followed by Tukey’s test was used for statistical analysis (ns, *P* > 0.05; *, *P* < 0.05; **, *P* < 0.01; ***, *P* < 0.001).

When HK senses an external signal molecule, it activates its cognate RR through phosphotransfer, thereby regulating target gene expression.^7^ We have previously shown that tetrathionate triggers phosphorylation between TtrS (His397) and TtrR (Asp58), which is essential for TtrRS-dependent transcriptional activation of target genes (Figure 3A).^20^ To determine whether TtrRS mediates the repressive effect of host intestinal molecules on QS, and to test whether tetrathionate is the specific intestinal cue responsible for this repression, we introduced the P_*opaR*_-*lux* reporter into strains carrying phosphorylation-site substitutions (D58A in TtrR or H397A in TtrS) and measured bioluminescence upon exposure to luminal contents from the cecum and small intestine. Deletion of *ttrR* or *ttrS* abolished the inhibitory effect of cecal contents on P_*opaR*_-*lux* luminescence, and genetic complementation with wild-type *ttrR* or *ttrS* fully restored this inhibition (Figure 3D). Importantly, the single amino acid substitutions D58A and H397A, introduced into the native chromosomal loci, completely eliminated the cecal content-mediated inhibition (Figure 3D). In contrast, small intestinal contents still suppressed P_*opaR*_-*lux* luminescence in all tested strains (Figure 3D). Collectively, these results establish that TtrRS is necessary for tetrathionate-mediated QS repression. However, given that tetrathionate is not typically abundant in the healthy gut, we next sought to determine the origin of the tetrathionate signal detected in our cecal extracts.

It is well established that healthy mouse cecal contents contain abundant thiosulfate but virtually no tetrathionate, whereas intestinal inflammation can oxidize thiosulfate to tetrathionate.^30^ ^,^ ^31^ Since our extracts were obtained from non‑inflamed mice, we investigated whether the tetrathionate signal was generated by the bacteria themselves. Our previous work showed that TsdBA, a direct transcriptional target of TtrRS, functions as a thiosulfate dehydrogenase that oxidizes thiosulfate to tetrathionate.^20^ To test this hypothesis, we performed HPLC analyzes to quantify thiosulfate and tetrathionate levels in healthy mouse cecal extracts before and after bacterial incubation. We found that incubation of cecal extracts with WT *V*. *parahaemolyticus* led to a marked increase in tetrathionate, whereas the Δ*tsdBA* mutant failed to produce tetrathionate (Figure 3E). Consistent with this, exogenous thiosulfate significantly repressed P_*opaR*_-*lux* activity in the WT strain, but this repression was strongly attenuated in both Δ*ttrR* and Δ*tsdBA* mutants (Figure 3F). This indicates that the thiosulfate‑mediated effect requires its conversion to tetrathionate via TsdBA and subsequent sensing through TtrRS. To further validate this model under physiologically relevant conditions, we incubated the same reporter strains with healthy cecal extracts. Cecal contents strongly repressed P_*opaR*_-*lux* luminescence in the WT strain, whereas this repression was largely abrogated in the Δ*ttrR* and Δ*tsdBA* mutants (Figure 3G). RT‑qPCR further confirmed that *opaR* mRNA levels correlated with the reporter data across all strains tested, including phosphorylation‑site point mutants and complemented strains (Figure S2). Collectively, these results demonstrate that *V*. *parahaemolyticus* converts host‑derived thiosulfate into tetrathionate via TsdBA, and that the newly generated tetrathionate activates TtrRS to repress QS, even in the absence of intestinal inflammation.

### TtrRS represses quorum sensing through H-NS in *V*. *parahaemolyticus*

We have previously demonstrated that TtrRS directly regulates target gene expression by binding to the *ttrR* box.^20^ However, sequence analysis revealed that a canonical *ttrR* box is absent from the promoter region of *opaR*, which suggests that TtrRS employs a previously unrecognized mechanism to modulate QS in *V*. *parahaemolyticus*. The σ^70^ factor (group I of the σ^70^ family) is the most abundant and primary *σ* factor in bacteria, responsible for initiating transcription from many housekeeping promoters.^32^ The histone-like nucleoid structuring (H-NS) protein is a global transcriptional silencer that selectively acts on RNA polymerase associated with σ^70^ (Eσ^70^), maintaining target genes in a repressed state until appropriate signals are received.^33^ To explore how TtrRS represses QS, we individually deleted five predicted σ^70^-encoding genes and the *h-ns* gene, and examined the resulting mutants for altered QS activity. Notably, the Δ*h-ns* strain exhibited the lowest luminescence among all tested strains (Figure 4A). Subsequent RT-qPCR analysis revealed that the mRNA level of *h-ns* was significantly higher in the Δ*ttrR* strain than in the WT strain, and this increase was restored to near WT levels in the complemented strain CΔ*ttrR* (Figure 4B). These findings indicate that H-NS is involved in the TtrRS-mediated QS regulation.

**Figure 4.**
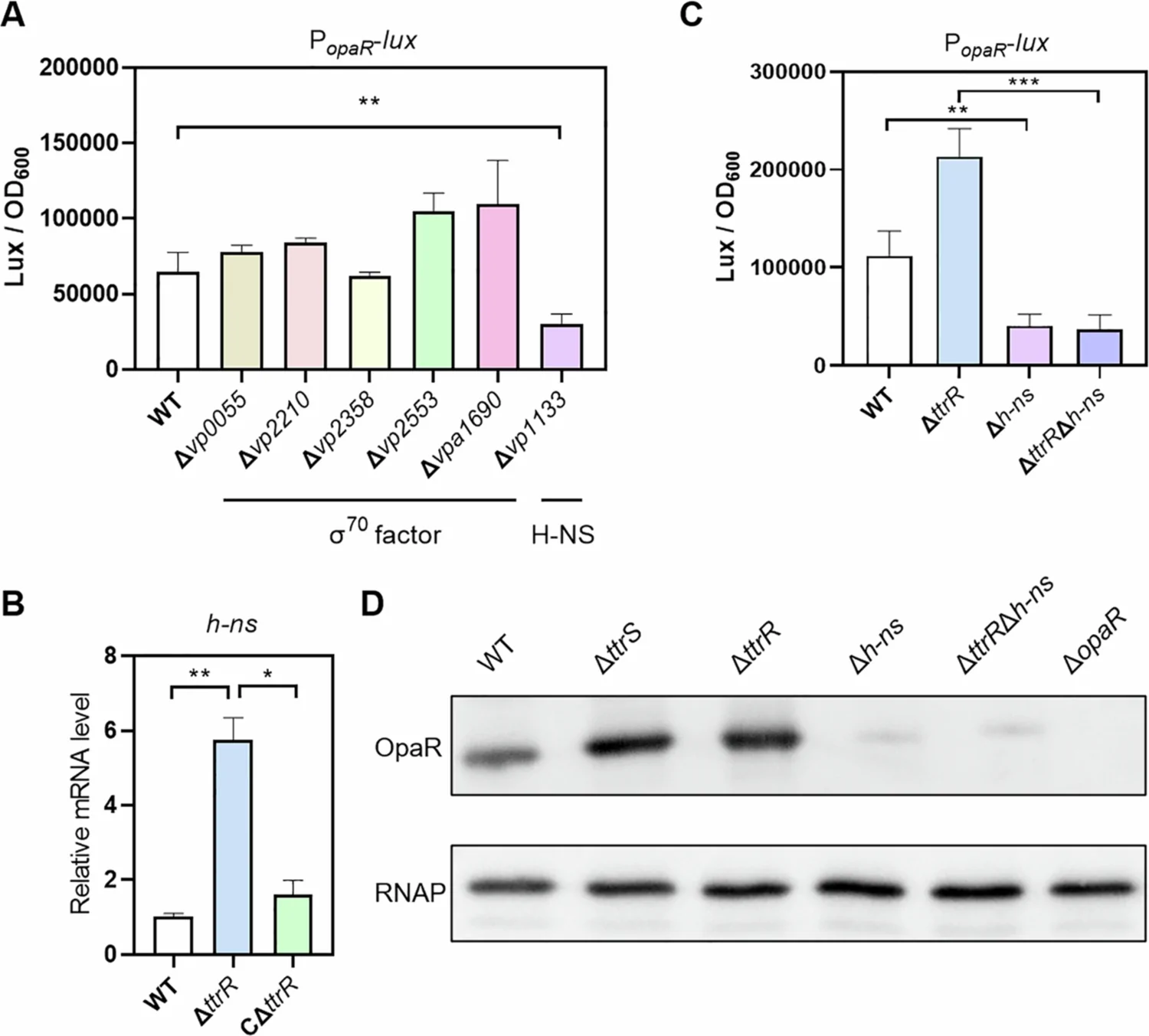
TtrRS represses QS through the global silencer H‑NS in *V. parahaemolyticus*. (**A**) Screening of mutants lacking predicted σ^70^‑encoding genes or *h‑ns* for altered QS activity. WT and individual deletion strains carrying the P_*opaR*_‑*lux* reporter were grown in MLB medium. Luminescence expression was calculated as the luminescence per unit of OD_600_. (**B**) RT‑qPCR analysis of *h‑ns* mRNA levels in WT, Δ*ttrR*, and CΔ*ttrR* strains. Total RNA was extracted from cultures grown to logarithmic phase, and *h‑ns* transcript abundance was normalized to the 16S rRNA gene. (**C**) P_*opaR*_‑*lux*‑driven luminescence in WT, Δ*ttrR*, Δ*h‑ns*, and Δ*ttrR*Δ*h‑ns* strains. Luminescence expression was calculated as the luminescence per unit of OD_600_. (**D**) Western blot analysis of OpaR protein levels in the indicated *V*. *parahaemolyticus* strains. Whole‑cell lysates were prepared from WT, Δ*ttrS*, Δ*ttrR*, Δ*h‑ns*, Δ*ttrR*Δ*h‑ns*, and Δ*opaR* strains. OpaR was detected using an anti‑OpaR antibody, with RNA polymerase (RNAP) serving as the loading control. The unpaired two-tailed Student’s *t*-test was used for statistical analysis (*, *P* < 0.05; **, *P* < 0.01; ***, *P* < 0.001).

To further characterize the role of H-NS in this regulatory axis, we constructed a Δ*ttrR*Δ*h-ns* double mutant and introduced the P_*opaR*_-*lux* reporter into WT and the relevant mutant backgrounds. Measurement of bioluminescence revealed that both the Δ*h-ns* single mutant and the Δ*ttrR*Δ*h-ns* double mutant displayed significantly reduced P_*opaR*_-*lux*-driven luminescence compared with the WT and Δ*ttrR* strains (Figure 4C). We next examined the effects of TtrRS and H-NS on OpaR protein production by western blotting. Deletion of *ttrS* or *ttrR* substantially increased OpaR expression relative to the WT strain, whereas deletion of *h-ns* markedly decreased OpaR expression (Figure 4D). Collectively, these results demonstrate that TtrRS represses QS activity in *V*. *parahaemolyticus* via H-NS.

### TtrRS and the quorum sensing master regulator OpaR exhibit cross-talk to co-regulate downstream genes and promote intestinal colonization

Both the TCS TtrRS and the QS master regulator OpaR are gene expression modulators, prompting us to explore their regulatory relationship. We previously performed transcriptomic analysis of Δ*ttrR* vs. WT *V*. *parahaemolyticus*, and the transcriptome of Δ*opaR* vs. *opaR*⁺ has been reported elsewhere.^20^ ^,^ ^34^ Comparative analysis of these two datasets revealed 76 genes that were differentially transcribed in both comparisons, predominantly in opposing directions: genes activated by TtrR were repressed by OpaR, and vice versa (Figure 5A,B). However, because these transcriptomes were generated in different strain backgrounds, the putatively co‑regulated targets required further experimental validation. To this end, we selected five TtrR‑activated and five TtrR‑repressed genes from the 76 overlapping candidates and measured their expression by RT‑qPCR in the WT, Δ*ttrR*, Δ*opaR*, and Δ*ttrR*Δ*opaR* double mutant strains, all cultured under identical conditions. In the single mutants, the expression patterns of all ten genes were consistent with the transcriptomic trends (Figure 5C), confirming the reliability of the original data. In the double mutant, however, two distinct behavior patterns emerged: some genes exhibited expression levels comparable to those in the Δ*ttrR* single mutant, while others resembled the Δ*opaR* single mutant (Figure 5C). This suggests that TtrR and OpaR do not operate in a simple linear hierarchy; instead, they converge on shared targets through independent or partially overlapping regulatory inputs.

**Figure 5.**
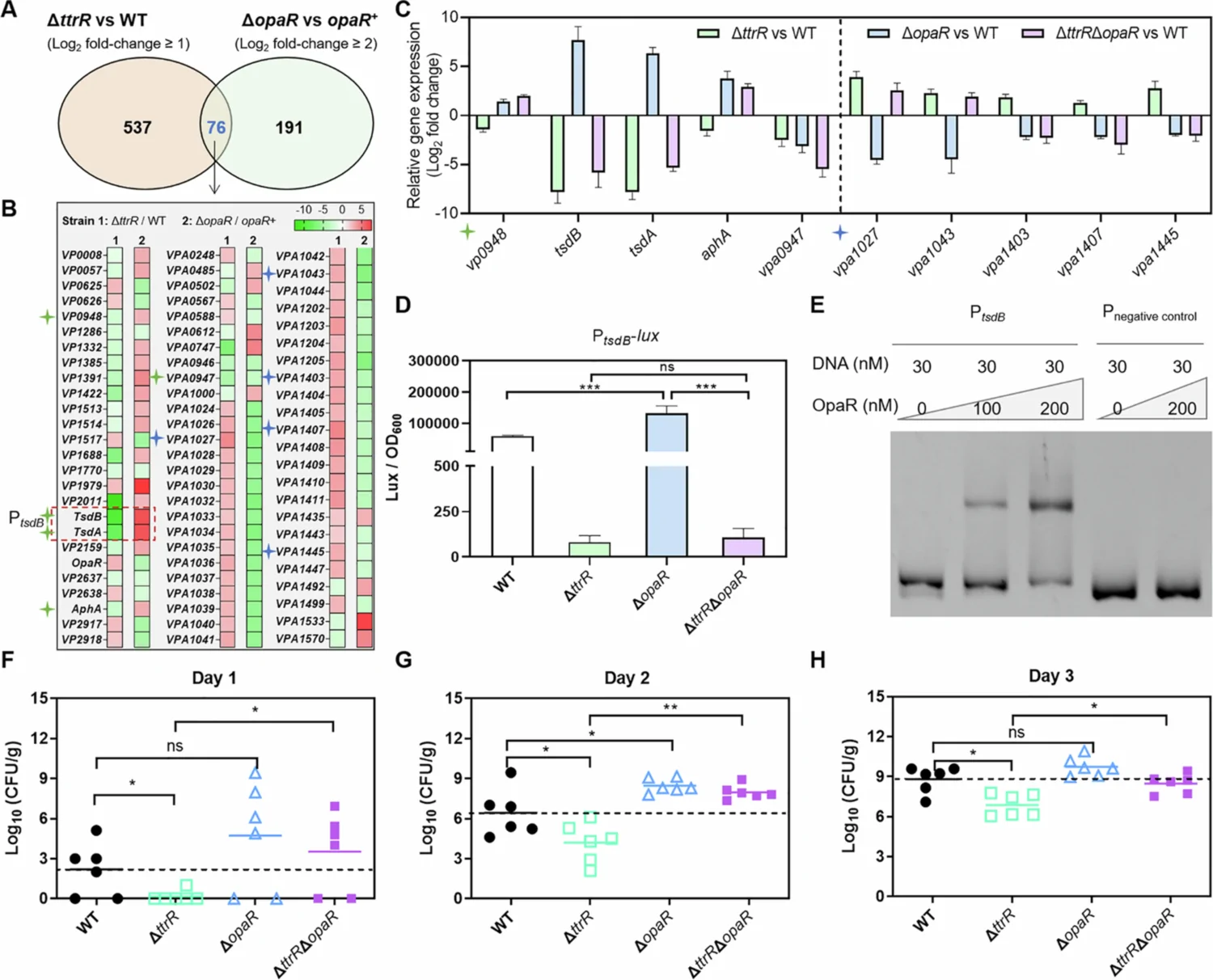
Cross‑talk between TtrRS and the QS master regulator OpaR in co‑regulating downstream genes and intestinal colonization. (**A**) Venn diagram showing the overlap of differentially expressed genes between the two transcriptomic datasets (Δ*ttrR* vs. WT and Δ*opaR* vs. *opaR*⁺). (**B**) Heatmap illustrating the expression patterns of the 76 co-regulated genes. The red dashed box highlights the *tsdBA* operon. (**C**) RT‑qPCR analysis of selected TtrR‑ and OpaR‑regulated genes in WT, Δ*ttrR*, Δ*opaR*, and Δ*ttrR*Δ*opaR* strains. Total RNA was extracted from cultures grown to logarithmic phase under identical conditions with 5 mM tetrathionate, and transcript levels were normalized to the 16S rRNA gene and then to the WT strain. Ten genes (five activated and five repressed by TtrR) were selected from the 76 overlapping candidates identified in the transcriptomic analysis for validation. (**D**) Effects of TtrR and OpaR on *tsdBA* promoter activity. A transcriptional reporter carrying the *tsdBA* promoter fused to the *lux* operon (P_*tsdB*_‑*lux*) was introduced into WT, Δ*ttrR*, Δ*opaR*, and Δ*ttrR*Δ*opaR* strains. Luminescence expression was calculated as the luminescence per unit of OD_600_. (**E**) EMSA analysis of OpaR binding to the *tsdBA* promoter. Purified OpaR protein was incubated with increasing amounts of a DNA fragment containing the *tsdBA* promoter region. OpaR bound the promoter in a dose‑dependent manner, whereas no binding was detected with a negative control fragment (16S rRNA gene). (**F–H**) Intestinal colonization of WT, Δ*ttrR*, Δ*opaR*, and Δ*ttrR*Δ*opaR* strains in a streptomycin‑pretreated mouse model. Colonization (CFU) of *V. parahaemolyticus* was measured from feces in the mouse model at the indicated time points. Groups containing six mice each were intragastrically administered with the indicated strains at a dose of 2.0 × 10^9^ CFU/mouse. The unpaired two-tailed Student’s *t*-test (D) or Mann-Whitney test (F–H) was used for statistical analysis (ns, *P* > 0.05; *, *P* < 0.05; **, *P* < 0.01; ***, *P* < 0.001).

Among the co‑regulated genes, *tsdBA*, a previously validated direct target of TtrRS, was also significantly upregulated in the Δ*opaR* transcriptome (Figure 5B). RT‑qPCR analysis revealed that *tsdBA* mRNA levels in the Δ*ttrR*Δ*opaR* double mutant were comparable to those in the Δ*ttrR* single mutant and significantly lower than in the WT strain (Figure 5C). Consistent with this, a transcriptional reporter carrying the *tsdBA* promoter (P_*tsdB*_-*lux*) showed that luminescence was substantially higher in the Δ*opaR* mutant than in the WT, but was almost completely abolished in both the Δ*ttrR* and Δ*ttrR*Δ*opaR* strains (Figure 5D). To further dissect the regulatory mechanism, we performed electrophoretic mobility shift assays (EMSA) using purified OpaR protein and a DNA fragment encompassing the *tsdBA* promoter. OpaR bound the *tsdBA* promoter in a dose‑dependent manner, whereas no binding was observed with a negative control fragment (Figure 5E). These results demonstrate that OpaR directly represses *tsdBA* transcription in a manner strictly dependent on functional TtrR, and further suggest that TtrRS and OpaR do not operate in a simple linear cascade, but instead engage in cross‑talk to co‑regulate downstream genes.

We have previously demonstrated that the TtrRS contributes to intestinal colonization by *V*. *parahaemolyticus.* ^20^ Given that TtrRS specifically senses tetrathionate generated from host-derived thiosulfate to repress QS, we next investigated whether QS participates in the TtrRS-mediated virulence of this pathogen. To address this, we employed a streptomycin-pretreated mouse model and monitored bacterial colonization by measuring bacterial loads in fecal samples. As shown in Figure 5F–H, mice infected with the Δ*opaR* strain exhibited significantly higher fecal bacterial loads than those infected with the WT strain. Similarly, the Δ*ttrR*Δ*opaR* double mutant colonized the mouse intestine to significantly higher levels than the Δ*ttrR* single mutant. These results indicate that both Δ*opaR* and Δ*ttrR*Δ*opaR* strains are better adapted to the gut environment than their respective controls. Furthermore, RT‑qPCR analysis revealed that *opaR* expression was strongly repressed in the WT strain during infection, whereas this repression was markedly attenuated in the Δ*ttrR* mutant (Figure S3). Collectively, our findings support a model in which *V*. *parahaemolyticus* senses tetrathionate (derived from cecal thiosulfate via TsdBA) through TtrRS to repress QS, thereby promoting intestinal colonization.

## Discussion

Successful intestinal colonization is a prerequisite for enteric pathogens to establish infection within the highly complex and dynamic host gut environment. During this process, pathogens must efficiently sense and respond to diverse external cues to enhance their colonizing capacity. TCSs and QS represent major signal transduction mechanisms that enable bacteria to adapt to fluctuating environmental conditions.^22^ ^,^ ^29^ ^,^ ^35^ ^,^ ^36^ In this study, we show that *V*. *parahaemolyticus* oxidizes host‑derived thiosulfate to tetrathionate via TsdBA, and that the resulting tetrathionate is sensed by the TtrRS system, which, through H‑NS, represses OpaR to dampen QS activity and promote intestinal colonization, revealing a novel regulatory axis that functions independently of the canonical autoinducer receptors (Figure 6). Our findings provide experimental evidence for a multi-system infection strategy employed by enteric pathogens, which may inform the development of antimicrobial agents targeting multiple bacterial signaling systems.

**Figure 6.**
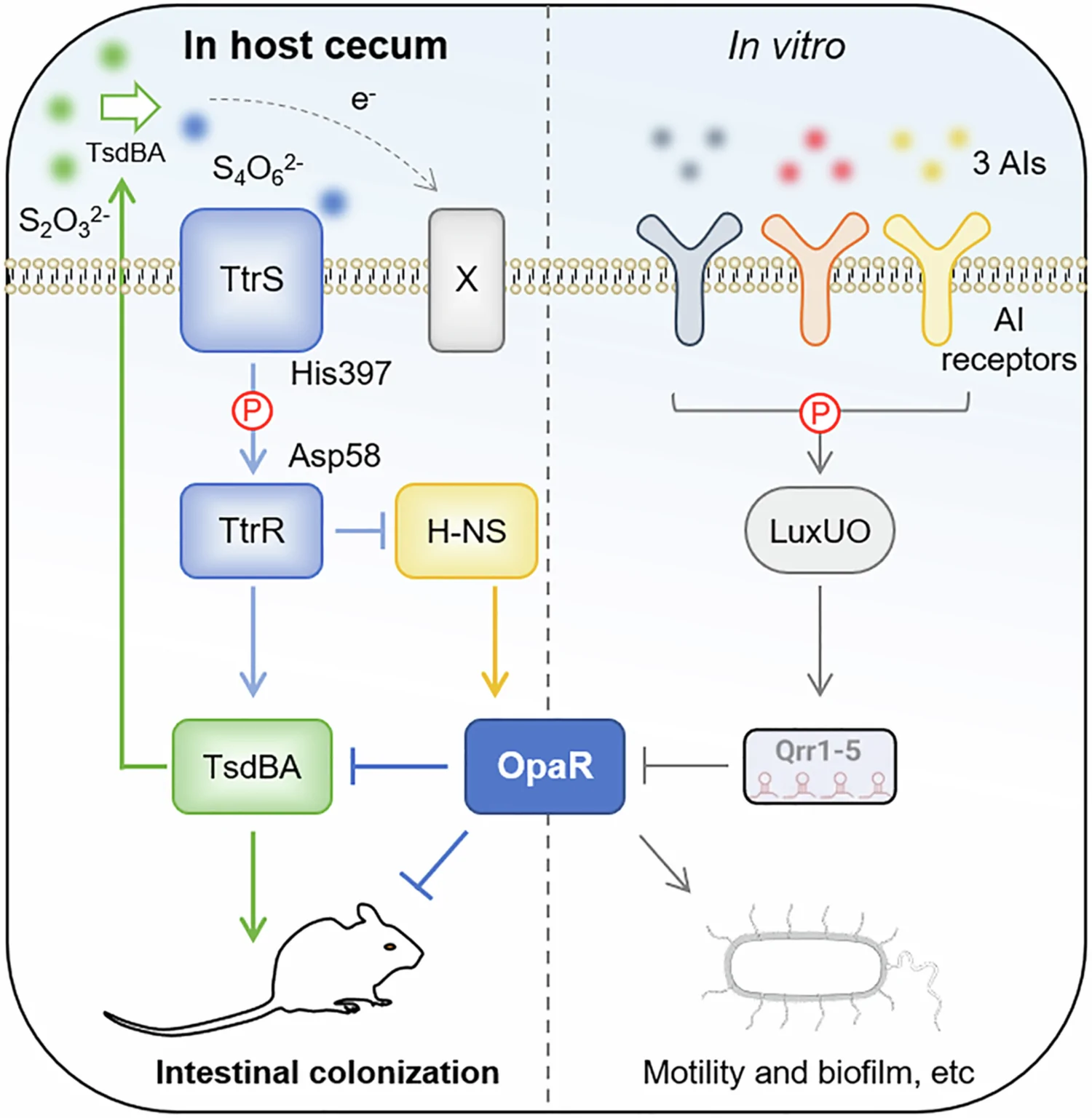
Proposed model of TtrRS-mediated QS repression during intestinal colonization. In the mouse cecum, *V*. *parahaemolyticus* oxidizes host-derived thiosulfate to tetrathionate via TsdBA. This oxidation releases electrons, which are transferred to an as-yet-unidentified electron acceptor (designated X, indicated by the dashed line). The newly generated tetrathionate is sensed by the membrane-bound histidine kinase TtrS, which subsequently phosphorylates the cognate response regulator TtrR at residue Asp58 through its conserved histidine residue His397. Activated TtrR directly upregulates *tsdBA* expression, reinforcing tetrathionate production in a positive feedback loop, while simultaneously repressing *h-ns* expression. Since H-NS acts as a positive regulator of the master QS regulator OpaR, TtrR-mediated repression of H-NS ultimately leads to reduced OpaR expression. In addition, OpaR directly binds the *tsdBA* promoter and represses its transcription, establishing a cross-regulatory loop with TtrR. In the absence of exogenous signals, this OpaR-mediated repression serves as a braking mechanism that prevents excessive TsdBA expression, thereby fine-tuning tetrathionate production and ensuring that the positive feedback loop is activated only when physiologically appropriate. Functionally, TtrR promotes intestinal colonization, whereas OpaR exerts an opposing effect by restricting colonization fitness. *In vitro*, QS is regulated through the canonical autoinducer (AI) receptor cascade, in which AIs bind their cognate receptors (LuxN, CqsS, and LuxPQ) to modulate the LuxU-LuxO phosphorelay and downstream Qrr1‑5 small RNAs, thereby controlling OpaR expression and QS-associated phenotypes such as motility and biofilm formation.

QS systems regulate pathogenicity in diverse bacterial species. In *Staphylococcus aureus*, the accessory gene regulator (*agr*) QS system functions as a key global regulator, orchestrating a temporal switch from the production of surface-associated colonization factors to the secretion of a broad array of exoproteins and toxins during the post-exponential growth phase.^37^ ^,^ ^38^ In *Pseudomonas aeruginosa*, the Las and Rhl QS systems hierarchically induce extracellular proteases such as LasB, which cleaves host immune factors and matrix proteins (elastin, fibrin, collagen), thereby disabling immunity and promoting tissue destruction.^4^ ^,^ ^39^ However, the molecular mechanisms by which pathogens integrate host-specific signals to modulate QS activity remain largely unexplored. *V*. *parahaemolyticus* possesses a sophisticated QS cascade. At low cell density, low AI concentrations fail to engage membrane-bound receptors, leading to the phosphorylation of LuxU and LuxO and subsequent activation of the small regulatory RNAs Qrr1-5, which repress the expression of the master regulator OpaR.^22^ ^,^ ^40^ Concomitantly, the alternative master regulator AphA is activated and further negatively regulates OpaR, collectively rendering QS inactive. Conversely, at high cell density, OpaR is abundantly expressed and directly or indirectly regulates hundreds of genes expression.^34^ In the present study, we observed that mouse intestinal contents significantly suppressed *V*. *parahaemolyticus* QS activity in a dose-dependent manner. Notably, this suppression persisted in a mutant lacking all three AI receptors genes (Figure 2), indicating that the inhibitory signal is independent of the canonical AI signaling machinery. These findings reveal that enteric pathogens can exploit host-derived metabolites to circumvent their own QS circuitry, uncoupling QS from its conventional cell-density dependence and enabling a previously unappreciated mode of regulation that operates through alternative sensory pathways.

Further investigation identified tetrathionate as the specific cecal cue responsible for QS repression (Figure 3). However, under homeostatic conditions, the healthy mouse gut contains abundant thiosulfate but little to no tetrathionate, as confirmed by our own HPLC analyzes. Tetrathionate is a byproduct of intestinal inflammation and microbial metabolism.^30^ ^,^ ^31^ Under homeostatic conditions, the gut microbiota produces hydrogen sulfide, which is detoxified by colonic epithelial cells to thiosulfate.^30^ ^,^ ^31^ During intestinal inflammation, the neutrophil respiratory burst oxidizes thiosulfate to tetrathionate.^30^ During gut colonization, *Salmonella typhimurium* exploits tetrathionate as a respiratory electron acceptor, thereby gaining a competitive advantage over the resident microbiota.^30^ Our previous work demonstrated that *V*. *parahaemolyticus* can oxidize thiosulfate to tetrathionate via the thiosulfate dehydrogenase TsdBA.^20^ Here, we further show that *V*. *parahaemolyticus* utilizes TsdBA to convert thiosulfate present in healthy cecal contents into tetrathionate, which is then sensed by TtrRS to repress QS. In many bacterial systems, thiosulfate dehydrogenases such as TsdA transfer electrons to the respiratory chain, often via cytochromes c or quinones.^41-43^ For *V*. *parahaemolyticus* TsdBA, however, the physiological electron acceptor has not yet been experimentally defined. Based on the general mechanism of thiosulfate dehydrogenases and our previous characterization of TsdBA,^20^ we speculate that it may involve components of the respiratory chain, potentially cytochrome c or a quinone pool, but this remains to be determined. Notably, this TsdBA‑dependent conversion occurs even in standard M9 or MLB media without exogenous thiosulfate supplementation, as TtrR‑dependent regulation of target genes is still observed under these conditions^20^ (Figure 3), indicating that the bacteria can generate tetrathionate from background sulfur compounds. Thus, we reveal a novel regulatory link between tetrathionate sensing and QS modulation in an enteric pathogen. These findings expand the known functions of tetrathionate from a respiratory substrate to a signaling molecule that represses QS, highlighting a previously unappreciated role for host-derived metabolites in directly shaping pathogen behavior.

We also observed that thiosulfate itself exerts a modest, TtrR‑independent repressive effect; however, this is unlikely to be the primary mechanism in the cecal environment, as the repressive effect of cecal contents was largely abolished in both Δ*ttrR* and Δ*tsdBA* mutants. Interestingly, the expression of *opaR* in the Δ*ttrR* mutant incubated with cecal contents was consistently lower than that of the WT strain in PBS, suggesting that additional TtrR‑independent signals may contribute to QS repression in the cecum. This possibility is supported by our observation that small intestinal contents repressed P_*opaR*_-*lux* luminescence in all tested strains, including those lacking TtrRS function (Figure 3D), indicating the presence of one or more TtrRS‑independent inhibitory molecules in the small intestine. It is therefore plausible that the cecum also harbors additional metabolites, whether host‑ or microbiota‑derived, that act through yet‑unidentified regulatory systems. Although our study focuses on the TtrRS‑tetrathionate axis as a major pathway for QS repression in the cecum, these residual effects highlight the complexity of QS regulation in the gut and suggest that multiple signaling inputs may converge to fine‑tune *V*. *parahaemolyticus* adaptation in different intestinal niches.

TCSs are conserved and versatile signal transduction modules that enable bacteria to sense external environmental cues and adapt to stressful conditions.^7^ In *V*. *parahaemolyticus*, several TCSs have been functionally characterized, playing diverse roles in antibiotic resistance, environmental adaptation, and virulence regulation.^18^ For instance, VbrK/VbrR senses *β*-lactam antibiotics and confers resistance to this class of antimicrobials;^44^ PeuRS responds to alkaline pH and exogenous iron, promoting bacterial survival under iron-limited conditions;^45^ and BarA/UvrY regulates the expression of T3SS and T6SS, thereby attenuating host innate immune responses and enhancing pathogenicity.^21^ The host intestine represents a critical niche for *V*. *parahaemolyticus* pathogenesis and serves as the primary battlefield for bacterial colonization. Although TCSs have been shown to participate in the pathogenic processes of various enteric pathogens within the host, whether they contribute specifically to intestinal colonization by *V*. *parahaemolyticus* remains to be determined. We previously reported that the TtrRS TCS senses tetrathionate and regulates the expression of its target genes *ttrBCA* and *tsdBA*, thereby contributing to intestinal colonization by *V*. *parahaemolyticus.* ^20^ ^,^ ^46^ In this study, we identify a novel regulatory axis in which TtrRS, upon sensing tetrathionate, represses QS via the global silencer H-NS (Figure 4). The H-NS is a multifunctional global transcriptional regulator in *V*. *parahaemolyticus* that plays a central role in virulence regulation, environmental adaptation, and biofilm formation by activating or repressing numerous genes.^47-52^ In our study, we found that H-NS promotes QS expression, a finding consistent with previous work showing that H-NS acts as a positive regulator of the master QS regulator OpaR in *V*. *parahaemolyticus.* ^49^ It was demonstrated that H‑NS does not directly bind the *opaR* promoter, yet a single H‑NS‑dependent promoter was identified with a transcription start site 74 bp upstream of *opaR*. This suggests that H‑NS activates *opaR* indirectly, likely through one or more unidentified intermediate factors. Our observation that H‑NS positively regulates OpaR is fully consistent with this established model, reinforcing the idea that H‑NS serves as a critical node connecting environmental sensing via TtrRS to QS output in *V*. *parahaemolyticus*.

In bacteria, crosstalk between distinct signal transduction systems can be achieved through multiple mechanisms, including shared response regulators, cross-phosphorylation, and co-regulation of target genes. A classic example is the *E*. *coli* nitrate-responsive NarX/NarL and NarQ/NarP TCSs, which exhibit asymmetric cross-regulation, thereby enhancing bacterial fitness under varying nitrate/nitrite conditions.^53^ In the present study, we found that TtrR and OpaR do not simply act in a linear pathway; rather, they converge on shared target genes through independent or partially overlapping regulatory inputs, which is a hallmark of cross-talk. This conclusion is supported by our observation that in a Δ*ttrR*Δ*opaR* double mutant, some co-regulated genes adopted expression patterns resembling those of the Δ*ttrR* single mutant, whereas others followed the Δ*opaR* single mutant (Figure 5). We further demonstrated that OpaR directly binds and represses the *tsdBA* promoter, providing direct molecular evidence that OpaR regulates a validated TtrR target. This regulatory relationship, in which a QS master regulator directly represses a TtrR-activated gene, exemplifies target gene co-regulation as a concrete form of cross-talk between the TtrRS and QS systems. Moreover, because TsdBA is the enzyme that generates tetrathionate from host-derived thiosulfate, and tetrathionate itself represses QS activity, these interconnections form a positive feedback loop that may fine-tune pathogen adaptation to the gut environment. In our model, TtrRS and QS appear to engage in cross-talk in response to specific environmental signals, a configuration that likely enhances the pathogen’s adaptability within the host gut. Consistent with this view, we investigated whether QS directly influences intestinal fitness. Although QS is well known to regulate virulence in many pathogens, its precise role in the adaptation of enteric pathogens to the gut environment has remained unclear. Our results directly show that deletion of the master QS regulator OpaR significantly improves *V*. *parahaemolyticus* colonization in the mouse gut (Figure 5), indicating that QS actively restricts intestinal fitness. Thus, by sensing tetrathionate through TtrRS and acting via H-NS, *V*. *parahaemolyticus* relieves this restriction, thereby promoting its colonization ability.

In summary, this study unveils a previously unrecognized mechanism of QS regulation. During intestinal infection, *V*. *parahaemolyticus* senses self‑generated tetrathionate (derived from host thiosulfate via TsdBA) via the TtrRS TCS and, through H-NS, represses QS activity to promote gut colonization. This regulatory cascade involves multiple critical regulators and confers a colonization advantage through potential cross-talk among different signaling modules. However, the precise molecular interactions among TtrRS, H-NS, and the QS network, as well as the detailed nature of their cross-talk, remain to be fully elucidated in future investigations.

## Materials and methods

### Bacterial strains, plasmids, and culture conditions

The bacterial strains and plasmids utilized in this work are detailed in the supplementary Table S1. The streptomycin-resistant *V*. *parahaemolyticus* strains were routinely cultured at 37 °C in modified Luria-Bertani (MLB) broth supplemented with 3% NaCl or in M9 minimal medium. *Escherichia coli* DH5α was grown in Luria-Bertani (LB) broth at 37 °C. When required, the media were supplemented with 50 μg/mL streptomycin, 5 μg/mL chloromycetin, 10% (w/v) sucrose, or 0.02% arabinose.

### Ethics statement and mouse assays

All animal procedures were conducted in accordance with the Laboratory Animal Welfare and Ethics Committee of Zhejiang A&F University, China (approval No. ZAFUAC2024018), and followed the Chinese National Laboratory Animal Guideline for Ethical Review of Animal Welfare. This study is reported in accordance with the ARRIVE guidelines (https://arriveguidelines.org), and a completed ARRIVE checklist is provided as supplementary material. Four‑week‑old female specific‑pathogen‑free (SPF) ICR mice were obtained from Hangzhou Medical College and maintained in standard cages under SPF conditions.

To prepare intestinal luminal contents, mice were euthanized by CO_2_ inhalation followed by cervical dislocation, and then dissected. The small intestine and cecum were collected separately, weighed, and homogenized in sterile phosphate-buffered saline (PBS) at a ratio of 1 mL per gram of tissue using a tissue homogenizer. After centrifugation, the supernatant was collected and passed through a 0.22‑μm filter. The resulting filtrate was mixed with M9 medium at appropriate ratios for subsequent bacterial luminescence assays.

Mouse infection experiments were performed as described previously.^20^ Each group consisted of six mice, and four groups were used, giving a total of 24 mice per experiment. Mice were randomly allocated using a computer‑generated random number sequence. The randomization sequence was generated prior to the start of the experiment, and allocation was performed by an investigator who was not involved in the subsequent data collection and analysis. Fecal colony enumeration was performed by an investigator blinded to group allocation. To minimize potential confounders, all mice were housed under identical environmental conditions (temperature 20–26°C, humidity 40–70%, 12‑h light/dark cycle, and diet). Throughout the experimental period, drinking water was supplemented with 1% (wt/vol) streptomycin and 0.8% (wt/vol) sucrose. Mice were pretreated with streptomycin for three days, followed by a 4‑h fasting period before inoculation. Each mouse received an intragastric administration of 100 μL of 10% (wt/vol) NaHCO₃ to neutralize gastric acid. Five minutes later, the mice were challenged intragastrically with 100 μL of a *V*. *parahaemolyticus* suspension at a dose of 2.0 × 10^9^ CFU per mouse. Food was returned 2 h post‑infection. Fecal samples were collected at the indicated time points, serially diluted in PBS, and plated onto MLB agar containing 50 μg/mL streptomycin for colony enumeration.

### Bioluminescence reporter assay

The reporter plasmid pBBR‑*lux* was delivered into *V*. *parahaemolyticus* strains via conjugation. Strains carrying the bioluminescent construct were grown in fresh MLB medium or in M9 medium supplemented with intestinal luminal contents, sodium tetrathionate, or sodium thiosulfate, at 37 °C for 6 h. Both luminescence intensity and cell density (OD₆₀₀) were measured using a Bio‑Tek Synergy HT spectrophotometer. Luminescence expression was expressed as the ratio of luminescence units to OD₆₀₀. All experiments were performed in triplicate.

### Recombinant DNA techniques

In‑frame markerless deletion mutants of *V*. *parahaemolyticus* were generated using the suicide plasmid pDS132 as previously described.^54^ Briefly, the upstream and downstream homology arms of the target gene were cloned into pDS132 in *E. coli* DH5α. The resulting recombinant plasmid was then mobilized into *V. parahaemolyticus* via conjugation with *E. coli* DH5α λpir. Putative deletion mutants were confirmed by PCR and subsequently validated by DNA sequencing. The CΔ*ttrR* ^WT^ and CΔ*ttrR* ^D58A^ strains were constructed based on the Δ*ttrR* strain by replenishing *ttrR* or *ttrR* ^D58A^ to the gene native locus, while the CΔ*ttrS* ^WT^ and CΔ*ttrS* ^H397A^ strains were constructed based on the Δ*ttrS* strain by replenishing *ttrS* or *ttrS* ^H397A^ to the gene native locus. The mutant strains were further verified by sequencing.

For promoter activity analysis, the reporter plasmid pBBR‑*lux* was employed as reported earlier.^54^ The predicted promoter region of *opaR* was amplified by PCR using gene‑specific primers and inserted into pBBR‑*lux*. To achieve protein overexpression, the pBAD24 vector (carrying an arabinose‑inducible promoter) was used to express TtrR in *V*. *parahaemolyticus*. All constructed recombinant plasmids were propagated in *E*. *coli* for amplification and verified by sequencing. Primers for PCR amplification were designed with Clone Manager 9 and are listed in the supplementary Table S2. Restriction enzymes used in this study were purchased from New England BioLabs and APExBIO.

### High-performance liquid chromatography analysis

To quantify thiosulfate and tetrathionate in cecal extracts before and after bacterial incubation, high‑performance liquid chromatography (HPLC) was employed.^20^ ^,^ ^55^ Cecal tissues were collected, weighed, and homogenized in sterile PBS. The homogenates were centrifuged, and the resulting supernatants were collected and passed through a 0.22‑μm filter. The filtrates were then mixed with M9 medium and incubated with the indicated *V*. *parahaemolyticus* strains for 9 h at 37 °C. After incubation, the bacterial cultures were centrifuged at 14,000 × g for 5 min. The cleared supernatant was mixed with 1 M Tris and 60 mM monobromobimane and incubated at room temperature for 10 min in the dark. Proteins were precipitated by the addition of metaphosphoric acid, and the mixture was centrifuged at 12,000 × g for 5 min at 4 °C. The concentrations of thiosulfate and tetrathionate in the samples were determined using SHIMADZU LC-16 system (Shimadzu, Japan). Standard chemicals including Na_2_S_2_O_3_ and Na_2_S_4_O_6_ were used to prepare the calibration curves for quantification of thiosulfate and tetrathionate.

### RNA extraction and RT-qPCR

*V*. *parahaemolyticus* strains were harvested either from the intestines of infected mice at 72 h post‑inoculation, or from cultures grown to logarithmic phase in MLB or M9 medium supplemented with sodium tetrathionate or cecal luminal contents at 37 °C. Cells were collected and washed three times with ice‑cold PBS. Total RNA was extracted using TRIzol reagent (Vazyme, China) according to the manufacturer’s instructions. RNA purity and concentration were assessed spectrophotometrically by measuring the A₂₆₀/A₂₈₀ ratio (≥1.8) using a Nanodrop 2000 (Thermo Fisher Scientific). After removal of contaminating genomic DNA, complementary DNA (cDNA) was synthesized from the RNA template using the HiScript II 1st Strand cDNA Synthesis Kit (Vazyme). Quantitative PCR was performed on an Mx3000P system (Agilent Technologies) with ChamQ Universal SYBR qPCR Master Mix (Vazyme). The 16S rRNA and *recA* genes were used as an internal reference for normalization.^40^ ^,^ ^56^ Primer sequences are listed in Table S2. Relative transcript abundance was calculated using the 2^−ΔΔCT^ method.^57^ Each reaction was run in triplicate.

### Western blot analysis

*V*. *parahaemolyticus* strains were inoculated into M9 medium supplemented with 5 mM sodium tetrathionate and grown to logarithmic phase at 37 °C. Cells were harvested by centrifugation, and total protein lysates were prepared by resuspending the pellets in a 3 × loading buffer. After denaturation at 90 °C for 30 min, a fraction containing 5 μg of total protein per lane was loaded and separated via SDS-PAGE. Separated proteins were electrophoretically transferred onto PVDF membranes (Millipore) using a wet transfer system (Bio-Rad) at 100 V for 1 h. Membranes were blocked with 5% (w/v) non-fat milk in TBST (Tris-buffered saline with 0.1% Tween-20) for 2 h at 37 °C with gentle shaking (50 rpm). The membrane was incubated for 2 h at 37 °C with the primary antibody against OpaR^58^ or RNA polymerase (RNAP). The RNAP antibody was raised in New Zealand white rabbits using recombinant RNAP protein expressed from pET28a in *E*. *coli*. After three washes with TBST (10 min each), the membranes were incubated with the secondary antibody (horseradish peroxidase-conjugated, Beyotime), followed by three additional washes. Chemiluminescent signals were generated using Immobilon® HRP substrate (Millipore) and captured on a Minichemi™ imaging system (Sagecreation). Each experiment was repeated at least three times.

### Electrophoretic mobility shift assay

To assess the direct interaction between OpaR and the target promoter, electrophoretic mobility shift assays (EMSAs) were conducted. The coding sequence of *opaR* was cloned into pET28a and expressed in *E*. *coli* BL21(DE3) following induction with 0.1 mM isopropyl‑β‑D‑thiogalactopyranoside (IPTG, Sigma‑Aldrich) at 16 °C for 10 h. Cells were harvested by centrifugation, disrupted by sonication, and the cleared lysate was loaded onto a Ni‑nitrilotriacetic acid spin column (GE Healthcare) for affinity purification according to the manufacturer’s recommendations. The eluted protein was dialyzed overnight at 4 °C and its purity was verified by SDS‑PAGE. DNA probes containing the *tsdBA* promoter region were generated by PCR and purified using a gel extraction kit (Vazyme). For binding assays, increasing amounts of purified OpaR protein were incubated with a fixed concentration of the DNA probe in binding buffer^20^ at 37 °C for 30 min. The reaction mixtures were then resolved on a 6% native polyacrylamide gel in 0.5 × TBE buffer at 200 V for 45 min on ice. After electrophoresis, the gel was stained with 1 × SYBR Gold nucleic acid stain (Invitrogen) in 0.5 × TBE buffer for 20 min, and images were captured using a gel documentation system.

### Statistical analysis

Data are expressed as mean ± standard deviation (SD). Statistical analyzes were conducted using an unpaired two-tailed Student’s *t* test, a one-way ANOVA followed by Tukey’s test, or Mann-Whitney test with the GraphPad software package. For all tests, a *P* value < 0.05 was considered statistically significant.

## Supplementary Material

Supplementary Figures S1 to S3.docx Supplementary Figures S1 to S3.docx

Supplementary Table S2.docx Supplementary Table S2.docx

Supplementary Table S1.docx Supplementary Table S1.docx

## Data Availability

The datasets used and/or analyzed during the current study are available at Figshare under the identifier DOI: 10.6084/m9.figshare.33372625.
