# Supplementary material for: Tetrathionate sensing enables Vibrio parahaemolyticus to repress quorum sensing for intestinal colonization
Source: Gut Microbes. 2026 Oct 15;18(1):2748145. doi: 10.1080/19490976.2026.2748145 (PMC13679331; doi:10.1080/19490976.2026.2748145)
Supplement: Supplementary file 1 [file KGMI_A_2748145_SM8292.docx]

**Table S2** Primers used in this study

| **Primer** | **Primer sequence (5'–3')** |
| --- | --- |
| pDS132-*vp0538* | P1: GCTCTAGAATGTTCGCCATGAAGTGGTACATAACCGTT |
|  | P2: CTGGCAGAGCTCATTCCAGTAAGCCGTCGT |
|  | P3: GCTTACTGGAATGAGCTCTGCCAGCTCTTC |
|  | P4: ACATGCATGCGCATTGGCGATGACCACCATAGACCGAATA |
| pDS132-*vp0569* | P1: ACATGCATGCGCATGCCAGTTAAGCGCAAGTTTCGTCACGACTTTG |
|  | P2: CAGTTTGTCGTGGAAGCAAAGCATTTCACG |
|  | P3: ATGCTTTGCTTCCACGACAAACTGATTCAAA |
|  | P4: AACTGCAGAACTTGAGTTTATGTTGGTCATCACCACTT |
| pDS132-*vp0914* | P1: GCTCTAGAGTGTACTCATTCTTGATCGCGAACTTTATA |
|  | P2: ATAGAACAAACGCGAAAAACGCTGTATGAC |
|  | P3: CAGCGTTTTTCGCGTTTGTTCTATTGCAAATCG |
|  | P4: ACATGCATGCATATCGTTGTGCTTACGTTGGCCTTTACGG |
| pDS132-*vp1202* | P1: GCGGAGCTCTTGCTGCCAACAATGTGGGT |
|  | P2: TTTATGAATCAAAACAGGCACTTCATCGTCAAC |
|  | P3: TGAAGTGCCTGTTTTGATTCATAAAGTAGGGCA |
|  | P4: GCGGAGCTCGTCCGCACCTTAATTAGAA |
| pDS132-*vp1711* | P1: GCTCTAGATGGTCTAAACGTGTTGCCAGCTTGCTGTAA |
|  | P2: ACGCGAGTGATGATCATTGAACGTTGTTATAAAAAAGTGATA |
|  | P3: TTTTTTATAACAACGTTCAATGATCATCACTCGCGT |
|  | P4: CGAGCTCGAGTACTATCAAAGAAGGTGTACTCAGCAT |
| pDS132-*vp1734* | P1: GCTCTAGAGCTCGACAGAGAGCTGTTCGCAACCGGAAT |
|  | P2: AATGTACCCCACACCACGTTTGTCATCTTCTACGAGTAA |
|  | P3: CTCGTAGAAGATGACAAACGTGGTGTGGGGTAC |
|  | P4: ACATGCATGCAAGACGCGTTCAGATAAACTGTCTACTTTA |
| pDS132-*vp1983* | P1: GCTCTAGAGGCTTTTGTCTTGAATCGTTGGCTCAACCC |
|  | P2: CTCCAAGCGGAAGCCGACATTATCTTCTACCAAAAGTACACG |
|  | P3: CTTTTGGTAGAAGATAATGTCGGCTTCCGCTTG |
|  | P4: CGAGCTCTAAATTCTAACGACTTTTCATCGGCTTGCG |
| pDS132-*vp2183* | P1: GCTCTAGATAATACCGATTTACTTAGATTGGCTCATCA |
|  | P2: CACAGTCAAACGGCAATGAATATTACTCGAGTTAATAGACTT |
|  | P3: ATTAACTCGAGTAATATTCATTGCCGTTTGACTGT |
|  | P4: ACATGCATGCTCTGAACAAGCACTGCGAACAACATTGAGT |
| pDS132-*vp2478* | P1: GCTCTAGATTGTGGTTGTCGGTGAAAACCGTTCCGGCG |
|  | P2: CAGTCTTTGCAATTTATCAAATTTCTGTTTTTTGTAGAATTTAAA |
|  | P3: TACAAAAAACAGAAATTTGATAAATTGCAAAGACTGCTC |
|  | P4: ACATGCATGCTTTTTTTAATATTGGCAAGCATAGCTGTTC |
| pDS132-*vpa0021* | P1: GCTCTAGAGCGGTCCGTACTACGTGCATGTGCTGGTGA |
|  | P2: AGTTTCGCCAACGAGATGATGCCGCAATAA |
|  | P3: CGGCATCATCTCGTTGGCGAAACTAAGTTG |
|  | P4: CGAGCTCAGCGGGCATGGATGTGGTTAATCGCATCGC |
| pDS132-*vpa0148* | P1: GCTCTAGACTCAGATGATTACCTCGATTTGTTAGGTGC |
|  | P2: TTTGATGTGCTGAACTACTTCTAAAAGCTC |
|  | P3: TTAGAAGTAGTTCAGCACATCAAAACGTTT |
|  | P4: CGAGCTCCTTCAAGATCGCTACGCTTGGTCTCTTTGT |
| pDS132-*vpa0183* | P1: GCTCTAGATCTACTGCATGGTTTGGCTCATTGAACCAG |
|  | P2: CTGATTTAGGGTCTGACGAACAATGTCATCA |
|  | P3: ATTGTTCGTCAGACCCTAAATCAGAAAATGCA |
|  | P4: ACATGCATGCGTATTCAGTGGCCTTTTTTATTACTTCACA |
| pDS132-*vpa0710* | P1: AACTGCAGGACAGGCCCATTTCCTTTAACTCACTTTCA |
|  | P2: TTTAACGATAAACCAACCAACTAATGTTAAATTGGG |
|  | P3: TTAGTTGGTTGGTTTATCGTTAAACCCGCT |
|  | P4: ACATGCATGCATCAGGATGGGCAGCCAATATTGGTTTACT |
| pDS132-*vpa0743* | P1: ACATGCATGCGCATGCGTGCAAATGAAGCAAAGTCATCGGCATGAT |
|  | P2: AGACTGGTTACCATTAAATACAACAACATG |
|  | P3: GTTGTATTTAATGGTAACCAGTCTGGAATC |
|  | P4: AACTGCAGTAAAGAACTTCTTCCTACGGTAAAAAATCC |
| pDS132-*vpa1276* | P1: ACATGCATGCTATTTCCTTAGTATGAGTTCATCG |
|  | P2: GTTTAAACACAACACGCCGTAAGTGATGTGTGCTGT |
|  | P3: ACAGCACACATCACTTACGGCGTGTTGTGTTTA |
|  | P4: AACTGCAGTGGGCTTCTTTGCAATCCGTATCTTT |
| pDS132-*vpa1538* | P1: GCTCTAGATGAGCTGTCTTCTTTCAGCG |
|  | P2: ACGAATATTTTGTTGGTTCAAAGTCCAGCTTTGGT |
|  | P3: GACCAAAGCTGGACTTTGAACCAACAAAATATTCGTTTTCG |
|  | P4: CGAGCTCATGGAAATGTAAGATATACTTTT |
| pDS132-*vpa1732* | P1: GCTCTAGAATGTAGTCTATTTGATTTTATACGCGCTGA |
|  | P2: AAACTTCGATTTGTCCAAGTCATCAACAAAGATAACTGC |
|  | P3: GTTATCTTTGTTGATGACTTGGACAAATCGAAGTTTAAG |
|  | P4: CGAGCTCACTGGGATTTTCAGTTCGCTAGATGTCATT |
| pDS132-*vp0055* | P1: GCTCTAGACTTTGCCTCTGATGAGATCATCTTTGAACC |
|  | P2: GTCTTTTTCGATTTGTTTATTACCAGACTG |
|  | P3: GGTAATAAACAAATCGAAAAAGACCTCAAG |
|  | P4: ACATGCATGCAGCACCATTTGCGCTAACAATCAACGAGTC |
| pDS132-*vp2210* | P1: GCTCTAGAAAGCGTCGGTATGGATGTTCATCCCCGTGC |
|  | P2: TTGTCCTCTCTTAGTATCCATTTTTAATATGGCCACAGGA |
|  | P3: GTGGCCATATTAAAAATGGATACTAAGAGAGGACAAAAAAA |
|  | P4: ACATGCATGCCTGGACGATAAATTGAGCTTTTGCTCAGTG |
| pDS132-*vp2358* | P1: GCTCTAGATGCCCCTAGACAGTCATTTGTTTGAAAGCT |
|  | P2: TCTTAACCGAGATTTATCCATGTAAGCGTT |
|  | P3: TACATGGATAAATCTCGGTTAAGACTCGCA |
|  | P4: CGAGCTCTTGCGTCGCTAGGAAACTTTGGACGAACCA |
| pDS132-*vp2553* | P1: GCTCTAGACCGCCCAACTCTAGTTAAAGACGCAGGTAT |
|  | P2: AACACAGTATCCAAAGTTAGGGTTTACGTCGACTTC |
|  | P3: GAAGTCGACGTAAACCCTAACTTTGGATACTGTGTTGCT |
|  | P4: ACATGCATGCGCGTCATATTGGTATTAGCTTTGTATTAGC |
| pDS132-*vpa1690* | P1: GCTCTAGAAATAAAAAATCGTAGGGACCGTATCTTATC |
|  | P2: TCGAGTCGCCACACGATAGATATCTTGGTA |
|  | P3: GATATCTATCGTGTGGCGACTCGATTATTC |
|  | P4: ACATGCATGCGCTCTTCCCCTTAGTATTTCGAGTGCTGAG |
| pDS132-*vp1133* (*h-ns*) | P1: GCTCTAGATAATGAAACAGGAGAATGAGAAAAATCTAT |
|  | P2: AATTGCAGAAGGCTCACGAGAAAATGCACG |
|  | P3: TTTTCTCGTGAGCCTTCTGCAATTCAAGAG |
|  | P4: CGAGCTCCGAAGGTCAAATGAACCAGCTTCATCCAGA |
| pDS132-*opaR* | P1: GCGTCTAGATCGCGATAGATTTAGGTTCAC |
|  | P2: TCTAGGTAAGCGCTTTGTTGCTTACGTTTAAGAG |
|  | P3: AGCGCTTACCTAGATATGCTTTGCATCTAC |
|  | P4: GCGGAGCTCAATCGACGAGCGCGGCTTCATCA |
| P*_opaR_*-*lux* | P1: GCGGAGCTCTAGTGTATCACCACGAAGAG |
|  | P2: GCGGGATCCATCCATTTTCCTTGCCATTT |
| pET28a-*opaR* | P1: CATGCCATGGCCATGGACTCAATTGCAAAGAGACCTAGAACT |
|  | P2: CCGCTCGAGGTGTTCGCGATTGTAGATGCAAAGCATATC |
| pET28a-*rnap* | P1: GTGCCGCGCGGCAGCCATATGCTACTACGTGCTATCTTCGGTGAAA |
|  | P2: ACCAGTCATGCTAGCCATATGTTATGGGTTAAGTACGATGTCTACAGG |
| *opaR*-RT-qPCR | P1: CTCGCGAAGATTTGGTTGATG |
|  | P2: GCGTGTATGTCTAGGTCGATATT |
| *h-ns*-RT-qPCR | P1: CCGTAGCCTTCGTGCATTT |
|  | P2: CGACGCTCTTCAACAACAGTAG |
| 16s rRNA-RT-qPCR | P1: ACCGCCTGGGGAGTACGGTC |
|  | P2: TTGCGCTCGTTGCGGGACTT |
| *recA*-RT-qPCR | P1: GCTAGTAGAAAAAGCGGGTG |
|  | P2: GCAGGTGCTTCTGGTTGAG |
| *vp0948*-RT-qPCR | P1: CGAAGGAGAAGAGTTATGACGTTAG |
|  | P2: AAACACGGTGAACCAAATGAAG |
| *tsdB*-RT-qPCR | P1: CTGGTCTGAACGCGAATTATCT |
|  | P2: GCAGGATCGCTAAGCTCTTT |
| *tsdA*-RT-qPCR | P1: GCTTACGACTCACCCGAAAT |
|  | P2: ACCGCTTCATCGTTCATACC |
| *aphA*-RT-qPCR | P1: CTCTGCTAGCATCGGTTACTTC |
|  | P2: GGTTTGCCTTCTTGAGGTTCTA |
| *vpa0947*-RT-qPCR | P1: GGATGTAGCAGCAATGAAAGG |
|  | P2: CAGTTGGCACAACACCATTAG |
| *vpa1027*-RT-qPCR | P1: AGCGACAGCGGAACAATATAA |
|  | P2: CTTCACGGTCCATTGCTGTA |
| *vpa1043*-RT-qPCR | P1: CAAGTGATGAGCATGCAAAGG |
|  | P2: GCCGTGAGTCCCAGAAATAA |
| *vpa1403*-RT-qPCR | P1: GATGCCAATGCATGCTAAAGAG |
|  | P2: GAGAATGGAGCAGGTTGTAAGT |
| *vpa1407*-RT-qPCR | P1: GAGTCACATGCTCAAACGTAAAC |
|  | P2: TCTGCCATCCAGAAAGGTAAAG |
| *vpa1445*-RT-qPCR | P1: GGTCTTCTCGGCGACAATAAA |
|  | P2: CTGTCACTGCCACCAACTAAA |
| *tsdB*-EMSA | P1: CCGAGAAGGGAAAGGCGTG |
|  | P2: GCTGACTCACTTTAATTGTT |
| 16S rRNA-EMSA | P1: AATGCCTAGGAAATTGCCCTG |
|  | P2: CGCCCATTGTGCAATATTCCC |
