# Supplementary material for: Tetrathionate sensing enables Vibrio parahaemolyticus to repress quorum sensing for intestinal colonization
Source: Gut Microbes. 2026 Oct 15;18(1):2748145. doi: 10.1080/19490976.2026.2748145 (PMC13679331; doi:10.1080/19490976.2026.2748145)
Supplement: Supplementary file 2 [file KGMI_A_2748145_SM8293.docx]

**Table S1** Bacterial strains and plasmids used in this study

| **Strains or plasmids** | **Description** | **Source** |
| --- | --- | --- |
| Bacterial strains |  |  |
| RIMD 2210633 | *V. parahaemolyticus*, wild-type (WT) | Our laboratory |
| Δ3AI receptors | Deletion mutant of *cqsS*, *luxN* and *luxPQ* with WT background | Our laboratory (1) |
| Δ*luxO* | Deletion mutant of *luxO* with WT background | Our laboratory (1) |
| Δ*vp0538* | Deletion mutant of *vp0538* with WT background | This study |
| Δ*vp0569* | Deletion mutant of *vp0569* with WT background | This study |
| Δ*vp0914* | Deletion mutant of *vp0914* with WT background | This study |
| Δ*vp1202* | Deletion mutant of *vp1202* with WT background | This study |
| Δ*vp1711* | Deletion mutant of *vp1711* with WT background | This study |
| Δ*vp1734* | Deletion mutant of *vp1734* with WT background | This study |
| Δ*vp1983* | Deletion mutant of *vp1983* with WT background | This study |
| Δ*vp2009* (*ttrR*) | Deletion mutant of *ttrR* with WT background | Our laboratory (2) |
| Δ*vp2183* | Deletion mutant of *vp2183* with WT background | This study |
| Δ*vp2478* | Deletion mutant of *vp2478* with WT background | This study |
| Δ*vpa0021* | Deletion mutant of *vpa0021* with WT background | This study |
| Δ*vpa0148* | Deletion mutant of *vpa0148* with WT background | This study |
| Δ*vpa0183* | Deletion mutant of *vpa0183* with WT background | This study |
| Δ*vpa0710* | Deletion mutant of *vpa0710* with WT background | This study |
| Δ*vpa0743* | Deletion mutant of *vpa0743* with WT background | This study |
| Δ*vpa1276* | Deletion mutant of *vpa1276* with WT background | This study |
| Δ*vpa1538* | Deletion mutant of *vpa1538* with WT background | This study |
| Δ*vpa1732* | Deletion mutant of *vpa1732* with WT background | This study |
| Δ*ttrA* | Deletion mutant of *ttrA* with WT background | Our laboratory (2) |
| Δ*tsdBA* | Deletion mutant of *tsdBA* with WT background | Our laboratory (2) |
| CΔ*ttrR*^WT^ | Complement of *ttrR* with ∆*ttrR* background | This study |
| CΔ*ttrR*^D58A^ | Asp58 of TtrR was changed to alanine on WT background | This study |
| Δ*ttrS* | Deletion mutant of *ttrS* with WT background | This study |
| CΔ*ttrS*^WT^ | Complement of *ttrS* with ∆*ttrS* background | This study |
| CΔ*ttrS*^H397A^ | His397 of TtrS was changed to alanine on WT background | This study |
| Δ*vp0055* | Deletion mutant of *vp0055* with WT background | This study |
| Δ*vp2210* | Deletion mutant of *vp2210* with WT background | This study |
| Δ*vp2358* | Deletion mutant of *vp2358* with WT background | This study |
| Δ*vp2553* | Deletion mutant of *vp2553* with WT background | This study |
| Δ*vpa1690* | Deletion mutant of *vpa1690* with WT background | This study |
| Δ*vp1133* (*h-ns*) | Deletion mutant of *h-ns* with WT background | This study |
| CΔ*ttrR* | ∆*ttrR* with the plasmid pBAD24-*ttrR* | This study |
| Δ*ttrR*Δ*h-ns* | Deletion mutant of *ttrR* and *h-ns* with WT background | This study |
| Δ*opaR* | Deletion mutant of *opaR* with WT background | This study |
| Δ*ttrR*Δ*opaR* | Deletion mutant of *ttrR* and *opaR* with WT background | This study |
| *E. coli* DH5α | Cloning host for maintaining the recombinant plasmids | Our laboratory |
| *E. coli* DH5α λpir | Mobilization of plasmids into *V*. *parahaemolyticus* | Our laboratory |
| *E. coli* BL21 (DE3) | Host for expressing the recombinant proteins | Our laboratory |
| Plasmids |  |  |
| pDS132 | Suicide vector for *V. parahaemolyticus* mutagenesis | Our laboratory |
| pDS132-*vp0538* | Derived from pDS132 used to knock out *vp0538* in WT | This study |
| pDS132-*vp0569* | Derived from pDS132 used to knock out *vp0569* in WT | This study |
| pDS132-*vp0914* | Derived from pDS132 used to knock out *vp0914* in WT | This study |
| pDS132-*vp1202* | Derived from pDS132 used to knock out *vp1202* in WT | This study |
| pDS132-*vp1711* | Derived from pDS132 used to knock out *vp1711* in WT | This study |
| pDS132-*vp1734* | Derived from pDS132 used to knock out *vp1734* in WT | This study |
| pDS132-*vp1983* | Derived from pDS132 used to knock out *vp1983* in WT | This study |
| pDS132-*vp2183* | Derived from pDS132 used to knock out *vp2183* in WT | This study |
| pDS132-*vp2478* | Derived from pDS132 used to knock out *vp2478* in WT | This study |
| pDS132-*vpa0021* | Derived from pDS132 used to knock out *vpa0021* in WT | This study |
| pDS132-*vpa0148* | Derived from pDS132 used to knock out *vpa0148* in WT | This study |
| pDS132-*vpa0183* | Derived from pDS132 used to knock out *vpa0183* in WT | This study |
| pDS132-*vpa0710* | Derived from pDS132 used to knock out *vpa0710* in WT | This study |
| pDS132-*vpa0743* | Derived from pDS132 used to knock out *vpa0743* in WT | This study |
| pDS132-*vpa1276* | Derived from pDS132 used to knock out *vpa1276* in WT | This study |
| pDS132-*vpa1538* | Derived from pDS132 used to knock out *vpa1538* in WT | This study |
| pDS132-*vpa1732* | Derived from pDS132 used to knock out *vpa1732* in WT | This study |
| pDS132-*ttrR*^WT^ | Derived from pDS132 for the complement of *ttrR* with ∆*ttrR* background | Our laboratory (2) |
| pDS132-*ttrR*^D58A^ | Derived from pDS132 used to make the *ttrR*^D58A^ variant | Our laboratory (2) |
| pDS132-*ttrS* | Derived from pDS132 used to knock out *ttrS* | Our laboratory (2) |
| pDS132-*ttrS*^WT^ | Derived from pDS132 for the complement of *ttrS* with ∆*ttrS* background | Our laboratory (2) |
| pDS132-*ttrS*^H397A^ | Derived from pDS132 used to make the *ttrS*^H397A^ variant | Our laboratory (2) |
| pDS132-*vp0055* | Derived from pDS132 used to knock out *vp0055* in WT | This study |
| pDS132-*vp2210* | Derived from pDS132 used to knock out *vp2210* in WT | This study |
| pDS132-*vp2358* | Derived from pDS132 used to knock out *vp2358* in WT | This study |
| pDS132-*vp2553* | Derived from pDS132 used to knock out *vp2553* in WT | This study |
| pDS132-*vpa1690* | Derived from pDS132 used to knock out *vpa1690* in WT | This study |
| pDS132-*vp1133* (*h-ns*) | Derived from pDS132 used to knock out *h-ns* in WT | This study |
| pDS132-*opaR* | Derived from pDS132 used to knock out *opaR* in WT | This study |
| pBAD24 | Arabinose-induced expression plasmid | Our laboratory |
| pBAD24-*ttrR* | Derived from pBAD24 used to overexpress TtrR protein | Our laboratory (2) |
| pET28a | His-tag expressing vector | Our laboratory |
| pET28a-*opaR* | pET28a inserted in-frame with the *opaR* gene for expressing OpaR | This study |
| pET28a-*rnap* | pET28a inserted in-frame with the *rnap* gene segment encoding RNAP residues 900-1100 | This study |
| pBBR-*lux* | The bioluminescence reporter | Our laboratory |
| P*_vh_*-*lux* | The bioluminescence reporter containing *luxCDABE* operon of *Vibrio harveyi* | Our laboratory (1) |
| P*_opaR_*-*lux* | pBBR-*lux* containing the promoter of *opaR* | This study |
| P*_tsdB_*-*lux* | pBBR-*lux* containing the promoter of *tsdB* | Our laboratory (2) |

**References**

1.	Liu F, Wang F, Yuan Y, Li X, Zhong X, Yang M. 2023. Quorum sensing signal synthases enhance Vibrio parahaemolyticus swarming motility. Mol Microbiol 120:241-257.

2.	Zhong X, Liu F, Liang T, Lu R, Shi M, Zhou X, Yang M. 2024. The two-component system TtrRS boosts Vibrio parahaemolyticus colonization by exploiting sulfur compounds in host gut. PLoS Pathog 20:e1012410.
