# Supplementary material for: Tetrathionate sensing enables Vibrio parahaemolyticus to repress quorum sensing for intestinal colonization
Source: Gut Microbes. 2026 Oct 15;18(1):2748145. doi: 10.1080/19490976.2026.2748145 (PMC13679331; doi:10.1080/19490976.2026.2748145)
Supplement: Supplementary file 3 [file KGMI_A_2748145_SM8294.docx]

**Supplementary Figures S1 to S3**

**Figure S1: Screening of RR deletion mutants for altered QS activity in *V. parahaemolyticus*.** Individual deletion mutants of 18 predicted RR genes were constructed and assessed for QS activity using the P*_vh_*‑*lux* reporter. Luminescence expression was calculated as the luminescence per unit of OD_600_. The unpaired two-tailed Student’s *t*-test was used for statistical analysis (**, *P* < 0.01).

**Figure S2: RT‑qPCR analysis of *opaR* expression in response to cecal contents.** *V. parahaemolyticus* strains were incubated with cecal extracts, with WT grown in M9 medium as a control. *opaR* transcript levels were normalized to 16S rRNA and expressed relative to the WT with cecal contents. Data represent mean ± SD from three independent experiments. The unpaired two-tailed Student’s *t*-test was used for statistical analysis (**, *P* < 0.01; ***, *P* < 0.001).

**Figure S3: TtrR is required for *opaR* repression during intestinal colonization.** *V*. *parahaemolyticus* strains were harvested from the intestines of infected mice at 72 h, and *opaR* transcript levels were measured by RT‑qPCR and normalized to *recA*. Data represent mean ± SD from three independent experiments.
